# Refractory Heart Failure in Noncompaction Cardiomyopathy Despite Guideline-Directed Therapy: A Case Series

**DOI:** 10.7759/cureus.103996

**Published:** 2026-02-20

**Authors:** Varshitha Tumkur Panduranga, Asher Gorantla, Nana Osei, Gitanjali Reddy, Saroj Yadav, Sabu John

**Affiliations:** 1 Internal Medicine, State University of New York Downstate Health Sciences University, Brooklyn, USA; 2 Cardiology, State University of New York Downstate Medical Center, Brooklyn, USA

**Keywords:** guideline-directed medical therapy, heart failure with reduced ejection fraction, implantable cardioverter-defibrillator, non-compaction cardiomyopathy, refractory heart failure

## Abstract

Noncompaction cardiomyopathy (NCCM) is a rare myocardial disorder characterized by excessive trabeculations and thinning of the compacted layer of the left ventricle. It is associated with heart failure, arrhythmias, thromboembolism, and sudden cardiac death. Patients with NCCM-related heart failure with reduced ejection fraction (HFrEF) are typically treated with standard guideline-directed medical therapy (GDMT). Here, we present three cases of patients with NCCM-related HFrEF who were managed with GDMT. Despite optimal treatment, left ventricular ejection fraction (LVEF) demonstrated minimal improvement, with no significant change in clinical outcomes. These cases illustrate the limited and inconsistent response of NCCM-related HFrEF to standard GDMT. Although some patients may experience partial reverse remodeling, the overall benefit of medical therapy remains variable. Patients with NCCM and HFrEF may demonstrate limited responsiveness to standard therapies, warranting close monitoring and early consideration of advanced interventions such as implantable cardioverter-defibrillators (ICDs), left ventricular assist devices (LVADs), or heart transplantation. Tailored therapeutic strategies and further research are needed to improve outcomes.

## Introduction

Noncompaction cardiomyopathy (NCCM) is an uncommon genetic cardiomyopathy characterized by abnormal ventricular wall architecture. Rather than a uniformly compact myocardium, the ventricular wall consists of a thin outer compacted layer and a thicker inner noncompacted layer with prominent trabeculations and deep intertrabecular recesses that communicate with the ventricular cavity but not with the coronary circulation [1]. NCCM most commonly affects the left ventricle and may progress to advanced heart failure. Additional complications include malignant arrhythmias, sudden cardiac death, and thromboembolic events [1]. Therefore, timely recognition using imaging modalities such as transthoracic echocardiography (TTE) and cardiac magnetic resonance (CMR) imaging is essential.

In patients with NCCM who develop heart failure with reduced ejection fraction (HFrEF), management generally parallels that of other etiologies of HFrEF and includes guideline-directed medical therapy (GDMT). This typically consists of angiotensin receptor-neprilysin inhibitors (ARNIs), angiotensin-converting enzyme (ACE) inhibitors, angiotensin receptor blockers (ARBs), beta-adrenergic blockers, mineralocorticoid receptor antagonists (MRAs), sodium-glucose cotransporter 2 (SGLT2) inhibitors, and diuretics. While these therapies improve outcomes in most forms of HFrEF, patients with NCCM may not derive similar benefit. In this case series, we present three patients with NCCM-related HFrEF who demonstrated minimal improvement despite optimized GDMT, highlighting the challenges of managing heart failure in this population and the need for individualized approaches beyond standard therapy.

## Case presentation

Case 1

History of Present Illness

A 58-year-old male presented to the emergency department with progressive shortness of breath and bilateral lower extremity swelling.

Past Medical History

**Figure 1:**
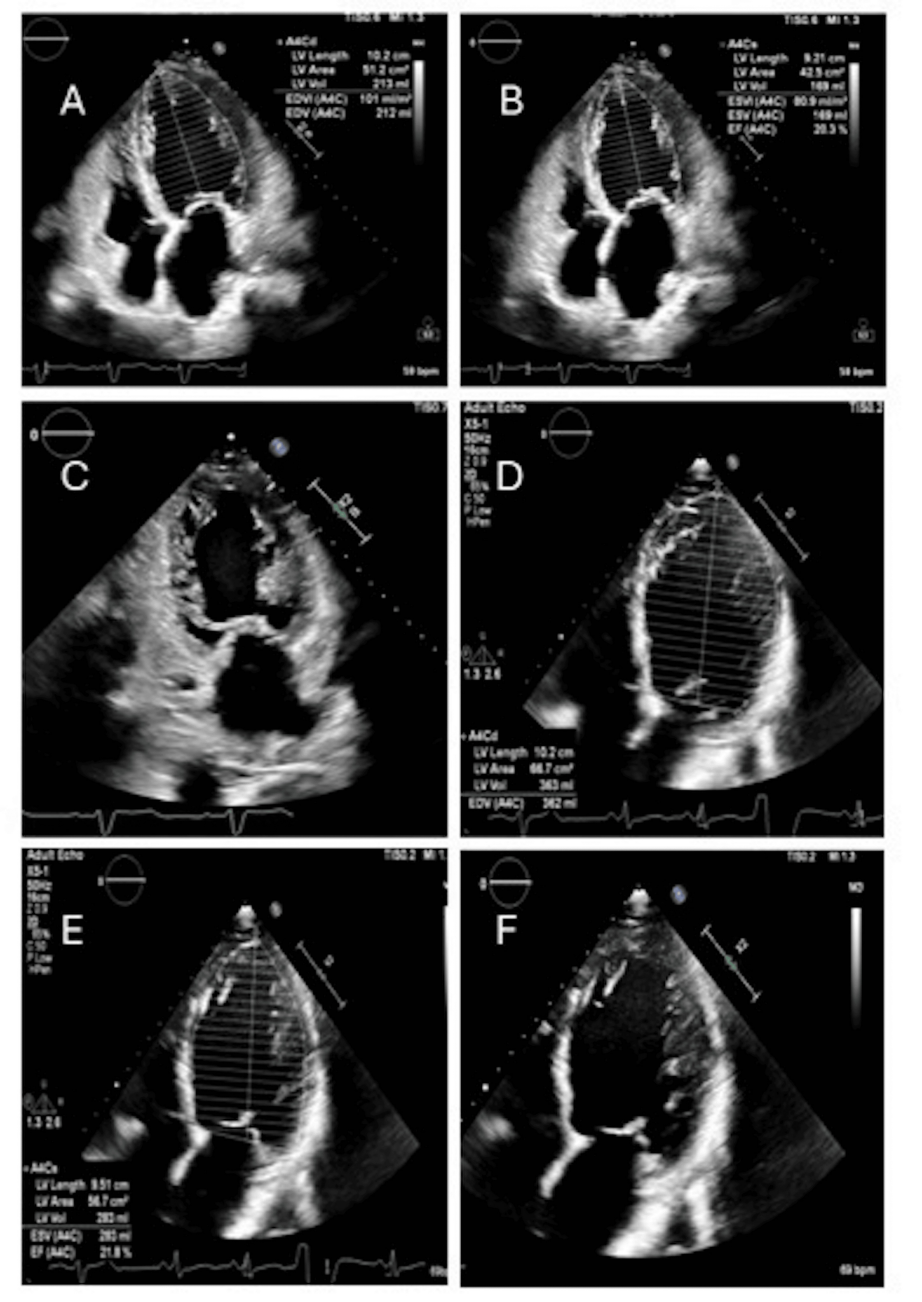
Transthoracic echocardiography (TTE) images for Case 1. (A-B) TTE with ejection fraction (EF) obtained by biplane/Simpson showing EF of 20.3%. (C) TTE showing trabeculations of the left ventricle (LV). (D-E) Repeat TTE showing EF of 21.8%, indicating no improvement. (F) TTE showing persistent trabeculations of the LV.

His medical history was significant for nonischemic cardiomyopathy, HFrEF with a left ventricular ejection fraction (LVEF) of 20.3% on TTE (Figures 1A-1B), NCCM (TTE demonstrating left ventricular trabeculations; Figure 1C), hypertension, paroxysmal atrial fibrillation, and type 2 diabetes mellitus. Home medications included sacubitril/valsartan, empagliflozin, and carvedilol (GDMT was already optimized).

Physical Examination

On presentation, blood pressure was 162/116 mmHg. Physical examination revealed bilateral pulmonary crackles and bilateral lower extremity edema.

Investigations

Laboratory evaluation showed an N-terminal pro-B-type natriuretic peptide (NT-proBNP) level of 18,000 pg/mL. Electrocardiography (ECG) demonstrated atrial fibrillation with rapid ventricular response. Thyroid function tests were within normal limits. Repeat TTE revealed persistently reduced LVEF of 21.8% (Figures 1D-1E) with prominent left ventricular trabeculations consistent with NCCM (Figure 1F) by Jenni criteria (ratio of compacted to non-compacted layer >2:1 in systolic phase).

Management and Clinical Course

The patient received intravenous furosemide in the emergency department and was admitted for acute decompensated HFrEF and atrial fibrillation with rapid ventricular response. Intravenous amiodarone was initiated and later transitioned to oral therapy, resulting in restoration of sinus rhythm. Volume status improved with diuresis, and the patient remained hemodynamically stable throughout hospitalization.

Outcomes and Follow-Up

He was referred to the electrophysiology service for evaluation and placement of an implantable cardioverter-defibrillator (ICD). 

Case 2

History of Present Illness

A 36-year-old male presented to the emergency department with dyspnea, orthopnea, and bilateral lower extremity swelling.

Past Medical History

**Figure 2:**
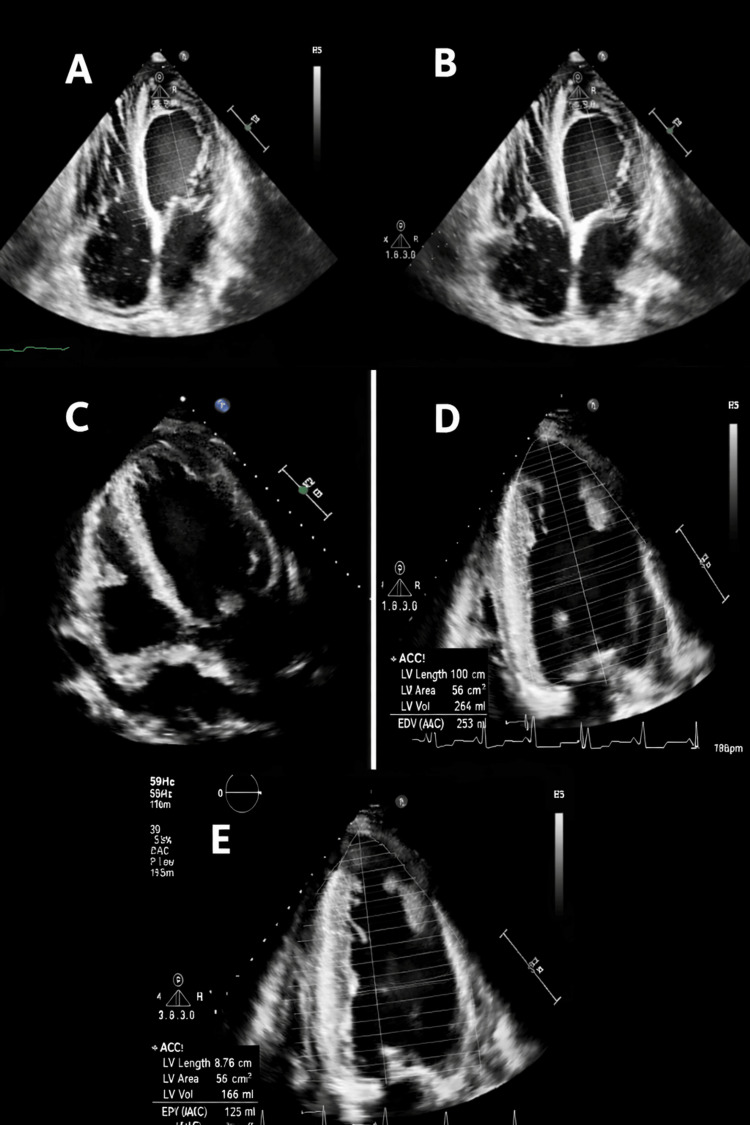
Transthoracic echocardiography (TTE) images from Case 2. (A-B) Parasternal short-axis views demonstrating prominent apical trabeculations consistent with noncompaction cardiomyopathy, along with a mild-to-moderate pericardial effusion. (C) Apical four-chamber view demonstrating a left ventricular ejection fraction (LVEF) of 35%. (D-E) Repeat TTE showing a reduced LVEF of 30.4%, indicating no significant improvement and a mild decline in systolic function.

His medical history was notable for NCCM (Figures 2A-2B, TTE showing apical trabeculations meeting Jenni's criteria for non-compaction cardiomyopathy), HFrEF with LVEF of 35% (Figure 2C), nonobstructive coronary artery disease, and end-stage renal disease (ESRD) on hemodialysis. Home medications included isosorbide dinitrate, hydralazine, and carvedilol (patient already on maximal tolerable doses of GDMT).

Physical Examination

On presentation, blood pressure was 218/113 mmHg with a heart rate of 92 beats per minute. Examination revealed bilateral pulmonary crackles and bilateral lower extremity edema.

Investigations

Laboratory studies demonstrated an elevated B-type natriuretic peptide (BNP) and a lactate level of 1.8 mmol/L. ECG showed normal sinus rhythm with left ventricular hypertrophy and no ischemic changes. TTE revealed a reduced LVEF of 30.4% (Figures 2D-2E), representing a decline from prior measurements.

Management and Clinical Course

The patient underwent hemodialysis and was initiated on bilevel positive airway pressure (BiPAP) ventilation, resulting in symptomatic improvement.

Outcomes and Follow-Up

He was referred to the electrophysiology service for evaluation for an ICD.

Case 3

History of Present Illness

A 61-year-old female presented with acute-onset dyspnea and bilateral lower extremity swelling.

Past Medical History

Her medical history included NCCM with a confirmed desmin (DES) gene mutation, HFrEF with an LVEF of 21% on TTE, and severe mitral regurgitation. Home medications included sacubitril/valsartan, spironolactone, furosemide, dapagliflozin, and bisoprolol (GDMT was optimized already). 

Investigations

**Figure 3:**
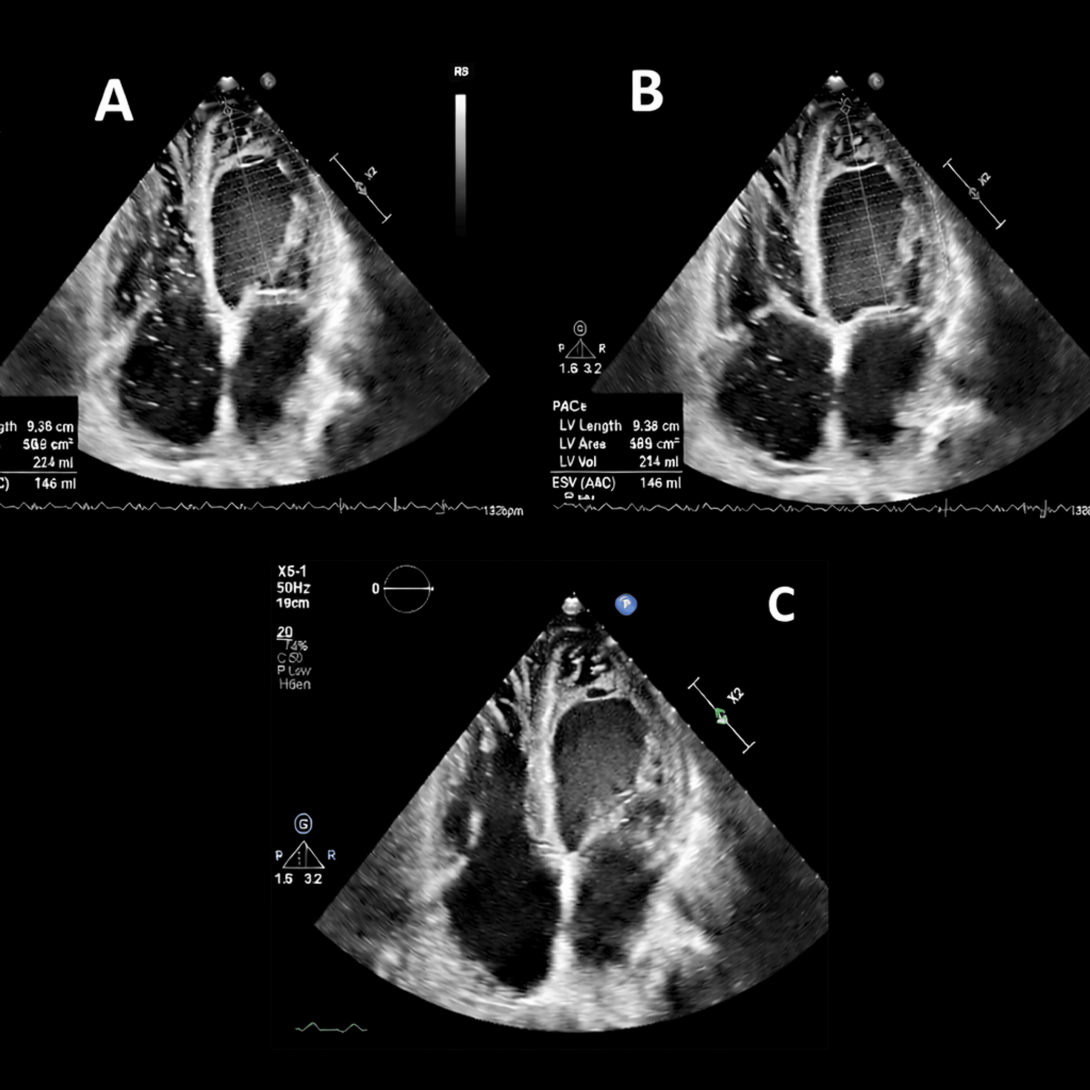
Transthoracic echocardiography (TTE) images from Case 3. (A-B) Apical four-chamber views demonstrating a severely reduced left ventricular ejection fraction (LVEF) of 4.46%. (C) Apical view showing prominent left ventricular (LV) trabeculations consistent with noncompaction cardiomyopathy.

TTE demonstrated a severely reduced LVEF of 4.46% with prominent left ventricular trabeculations consistent with NCCM (Jenni criteria) (Figures 3A-3C).

Management and Clinical Course

She was treated with noninvasive ventilation (BiPAP) and intravenous furosemide and admitted for acute decompensated heart failure. Over 24 hours, she achieved a net diuresis of 2 L with symptomatic improvement.

Outcomes and Follow-Up

She was discharged with a wearable cardioverter-defibrillator (LifeVest, ZOLL Medical Corporation, Chelmsford, MA). The patient was scheduled for an outpatient evaluation for ICD placement.

## Discussion

NCCM is increasingly recognized as a distinct cardiomyopathy with unique structural, genetic, and clinical features. Although GDMT remains the cornerstone of management for HFrEF, the cases presented here highlight the limitations of current treatment paradigms in NCCM. Despite optimized GDMT, including ARNIs, beta-blockers, MRAs, SGLT2 inhibitors, and diuretics, patients demonstrated persistent left ventricular dysfunction and limited clinical improvement.

The abnormal myocardial architecture of NCCM is characterized by a thin compacted epicardial layer and a thickened noncompacted endocardial layer with prominent trabeculations and deep recesses [1,2]. This structural phenotype contributes to impaired contractile efficiency, abnormal relaxation, and restricted coronary reserve, rendering the myocardium less responsive to neurohormonal blockade [3,4]. Additionally, myocardial fibrosis and microcirculatory dysfunction, consistently demonstrated with advanced imaging, may accelerate progression to end-stage heart failure independent of GDMT [4].

Genetic contributions are also central to disease expression. NCCM has been linked to pathogenic variants in sarcomeric, cytoskeletal, and mitochondrial genes [5]. In this series, one patient harbored a DES mutation, consistent with prior reports associating desmin mutations with aggressive phenotypes, increased arrhythmia burden, and progression to end-stage heart failure [6]. Recent genomic studies demonstrate that up to 25-90% of familial NCCM cases carry a pathogenic or likely pathogenic variant, highlighting the heterogeneity of the disease and the potential role of genotype in prognosis and therapeutic response [7-9].

These cases underscore a recurring theme: persistent dysfunction despite optimized GDMT. Observational studies confirm that patients with NCCM are at higher risk for adverse outcomes, including heart failure progression, malignant arrhythmias, and sudden cardiac death, compared with those with other cardiomyopathies [10,11]. Pediatric series demonstrate a particularly poor prognosis, with increased need for transplantation or advanced therapies [11]. In adults, registry analyses show that reduced LVEF is a key determinant of outcome, with patients in NYHA class III/IV experiencing the worst survival [5,12].

While GDMT may provide symptomatic improvement, the degree of reverse remodeling appears limited in NCCM. Parent et al. reported that medical therapy may promote favorable remodeling in select cases, although outcomes are inconsistent [13]. This aligns with the findings in this series, in which patients failed to achieve meaningful improvement despite maximal therapy. Moreover, recent data suggest that clinical risk prediction in NCCM remains challenging, and discontinuation or incomplete adherence to GDMT is associated with worse outcomes [14].

NCCM is associated with a high prevalence of atrial fibrillation, ventricular tachyarrhythmias, conduction disturbances, and systemic thromboembolic events [10,12,15]. One case in this series presented with atrial fibrillation and rapid ventricular response, reflecting this arrhythmic burden. Concomitant comorbidities, including chronic kidney disease, valvular regurgitation, and prior stroke, further complicated management. These factors may blunt the response to GDMT and accelerate disease progression, underscoring the need for a multidisciplinary approach to care.

Given the suboptimal outcomes observed with conventional therapy, escalation to advanced therapies should be considered early. Device-based interventions such as ICDs and cardiac resynchronization therapy (CRT) have demonstrated benefit in NCCM, particularly among patients with severe systolic dysfunction and arrhythmias [12]. In this series, two patients were discharged with wearable cardioverter-defibrillators, reflecting a heightened risk of sudden cardiac death. Mechanical circulatory support with left ventricular assist devices (LVADs) has been described in refractory cases. Ultimately, heart transplantation remains the definitive therapy in advanced disease, although genetic and systemic considerations may complicate patient selection. Case reports also suggest that SGLT2 inhibitors may offer incremental benefit in NCCM patients with HFrEF, though evidence remains limited [14].

This series illustrates several important lessons for clinicians. First, NCCM should be recognized as more than a morphological variant of dilated cardiomyopathy; it is a distinct entity with unique pathophysiologic drivers [2,3]. Second, clinicians should anticipate variable or absent responses to GDMT, necessitating early recognition of treatment resistance and timely referral for advanced heart failure care. Third, genetic testing is increasingly valuable, as it informs prognosis, guides family screening, and may ultimately enable genotype-directed therapies [5,7-9]. Finally, optimal management requires a multidisciplinary team incorporating expertise in advanced imaging, electrophysiology, heart failure, and cardiovascular genetics.

Although limited by a small sample size, this case series contributes to the growing body of evidence suggesting that NCCM differs fundamentally from other etiologies of HFrEF. Future research should focus on prospective registries, clinical trials evaluating GDMT efficacy in NCCM, and mechanistic studies linking genotype to phenotype. Emerging evidence supports the importance of precision medicine approaches, with ongoing efforts to characterize the prognostic impact of specific mutations and to evaluate novel therapeutic strategies [7-9,15,16].

## Conclusions

NCCM usually responds to guideline-directed medical therapy. However, in our three cases, the ejection fraction did not improve even after GDMT optimization. This observation supports the concept that NCCM follows a clinical trajectory distinct from other etiologies of HFrEF. Clinicians should maintain a high index of suspicion for treatment resistance and consider early referral for advanced therapies, including ICDs for primary prevention of sudden cardiac death, mechanical circulatory support with LVADs, and, ultimately, heart transplantation. Early recognition and individualized management are essential to improving outcomes in this high-risk population.
